# The Development and Validation of a Nomogram for Predicting Sepsis Risk in Diabetic Patients with Urinary Tract Infection

**DOI:** 10.3390/medicina61020225

**Published:** 2025-01-27

**Authors:** Hua-Qiao Tan, Xiang-Jie Duan, Wan Qu, Mi Shu, Guang-Yao Zhong, Li-Hong Liang, Dong-Mei Bin, Yu-Ming Chen

**Affiliations:** 1 Department of Epidemiology, School of Public Health, Sun Yat-Sen University, Guangzhou 510080, China; tanhq7@mail2.sysu.edu.cn (H.-Q.T.); shum7@mail2.sysu.edu.cn (M.S.); 2 Department of Intensive Care Unit, The Sixth Affiliated Hospital of Jinan University, Dongguan 523560, China; zhongguangyao87@163.com (G.-Y.Z.); lianglihong1120@163.com (L.-H.L.); bingdongmei51@163.com (D.-M.B.); 3 Department of Intensive Care Unit, The First Affiliated Hospital of Jinan University, Guangzhou 510632, China; duanxiangjie@stu2024.jnu.edu.cn; 4 Department of Health Management Centre, The First Affiliated Hospital of Jinan University, Guangzhou 510632, China; 18188805351@163.com

**Keywords:** diabetes mellitus, urinary tract infection, sepsis, MIMIC, risk factors, nomogram

## Abstract

*Background and Objectives*: Urinary tract infection (UTI) is a common comorbidity in diabetic patients, making up one of the causes of sepsis. This study aims to develop a nomogram to predict the risk probability of sepsis in diabetic patients with UTI (DPUTIs). *Materials and Methods*: This is a retrospective observational study. Clinical data for DPUTIs were extracted from the Medical Information Mart for Intensive Care IV database. Eligible DPUTIs were randomly divided into training and validation cohorts in a 7:3 ratio. Independent prognostic factors for sepsis risk were determined using least absolute shrinkage and selection operator (LASSO) regression and multivariate logistic regression. A corresponding nomogram based on these factors was constructed to predict sepsis occurrence in DPUTIs. The discrimination of the nomogram was assessed by multiple indicators, including the area under the receiver operating characteristic curve (AUC), net reclassification improvement index (NRI), and integrated discrimination improvement (IDI). In addition, a calibration curve and decision curve analysis (DCA) were used to evaluate the performance of the nomogram. *Results*: A total of 1990 DPUTIs were included. Nine independent prognostic factors were identified as predictive factors for sepsis risk in DPUTIs. The prognostic factors included urine red blood cell classification (urine RBC cat), urine white blood cell classification (urine WBC cat), blood glucose, age, temperature, white blood cells (WBCs), sequential organ failure assessment (SOFA) score, lymphocytes, and hematocrit. The AUC, NRI, and IDI of the nomogram indicated robust discrimination. The calibration curve and Hosmer–Lemeshow test showed good calibration of the nomogram. The DCA curve demonstrated a better clinical utility of the nomogram. *Conclusions*: The nomogram established in this study helps clinicians predict the probability of sepsis in DPUTIs, providing evidence for optimizing the management of related risk factors.

## 1. Introduction

Diabetic patients are prone to developing UTI, which may progress to sepsis without timely treatment [1]. As a clinical syndrome, sepsis is a life-threatening organ dysfunction due to a dysregulated host response to infection [2]. It is an important cause of death after deteriorating community-acquired or healthcare-associated infections, and 11.0 million sepsis-related deaths were reported in 2017 [3]. Sepsis-related death has become one of the global public health problems and remains a formidable challenge [4]. However, the etiology and determinants of sepsis secondary to UTI in diabetic patients have not been fully elucidated. Patients often need to receive intensive care or infection source control [5], which increases the medical burden. Therefore, the early identification of sepsis and appropriate treatments to block the progress of infection are very necessary. The early identification and elimination of sepsis risk factors in DPUTIs and the enhancement of management strategies may help reduce sepsis incidence.

Due to the large population of people with diabetes in the world, there are abundant patients with UTI each year, and studies have shown that UTI has become the most common cause of sepsis [6,7]. Previous studies have confirmed diabetes as an independent risk factor for infection [8], and the presence of diabetes may aggravate the condition of diseases [9]. Diabetes mellitus tends to be combined with multiple negative factors such as immune dysfunction, persistent positive urine glucose, the existence of organ dysfunction, and the coexistence of urolithiasis and may worsen the clinical condition of UTI patients, leading to urosepsis [10,11,12], which occurs not only in nosocomial infections but also in infections in the community or emergency departments [13]. The lack of early recognition of negative factors and corresponding treatments may be an obstacle to improving outcomes among these infected patients. Hence, DPUTIs should draw more attention to be effectively and fully evaluated. It is necessary to develop a tool to predict the risk of sepsis, which can be put into clinical practice early, especially in community hospitals. A nomogram is a graphical tool for determining the risk probability of clinical events, but it is rarely used to study the early warning risks of sepsis in DPUTIs. This study aimed to develop a nomogram for the early prediction of sepsis risk in DPUTIs to guide in-hospital or community interventions.

## 2. Materials and Methods

### 2.1. Data Sources

All DPUTI data were obtained from the Medical Information Mart for Intensive Care IV (MIMIC-IV) 3.0 database (version 3.0). The MIMIC-IV is a large, single-center, open-access critical care database established in 2003 with funding from the National Institutes of Health (NIH) of the United States. Version 3.0 (July 2024) contains medical data from 94,458 intensive care unit (ICU) admissions at Beth Israel Deaconess Medical Center (BIDMC) in Boston from 2008 to 2022. The data included patient demographic information, bedside vital signs, laboratory indicators, surgical records, disease diagnosis, survival data, etc. The research personnel finished a series of online courses required by the NIH and passed an examination for authorization to access the database (certificate number: 55333116). The data utilization of the MIMIC-IV database was approved by the Institutional Review boards of BIDMC and the Massachusetts Institute of Technology (MIT). Informed consent was not required for this study since the data were deidentified.

### 2.2. Study Population

Research data were extracted using PostgreSQL (version 16.3) software through Structured Query Language (SQL). The SQL script codes for extracting patient information were acquired from the GitHub website (https://github.com/MIT-LCP/mimic-code/tree/main/mimic-iv, accessed on 1 September 2024). Patients with diabetes were identified using International Classification of Diseases ninth and tenth revision (ICD-9 and ICD-10) codes. Patients with urinary tract infection (UTI) were identified based on microbiological culture results; those with positive urine culture results within 14 days prior to ICU admission were recognized as UTI patients. Data on patients with diabetes and UTI were extracted, with the outcome of interest being a diagnosis of sepsis (diagnostic criteria were based on the Sepsis-3 definition [2]) during the ICU stay. Exclusion criteria included the following: age < 18 years, ICU stay duration < 24 h, and repeated ICU admissions.

### 2.3. Data Collection

Clinical data extracted from the MIMIC-IV (version 3.0) for DPUTIs included demographic information, age, sex, race, height, and weight; disease severity scores assessed during the first 24 h after ICU admission, SOFA and acute physiology and chronic health evaluation II (APACHE-II) scores; urine culture results within the 14 days prior to ICU admission; initial laboratory examination results within the first 24 h after ICU admission, white blood cells, neutrophils, lymphocytes, hematocrit, platelets, red blood cell distribution width (RDW), blood glucose, blood urea nitrogen (BUN), creatinine, total bilirubin, urine red blood cells (urine RBCs), urine white blood cells (urine WBCs), urine pH, urine ketone, and urine protein; comorbidities, hypertension, congestive heart failure, chronic pulmonary disease, liver disease, renal disease, cerebrovascular disease, paraplegia, urinary obstruction, fluid electrolyte disorders, cancer, and urolithiasis; and initial vital signs within the first 24 h after ICU admission, respiratory rate, mean arterial pressure, temperature, and heart rate.

### 2.4. Statistical Analysis

Statistical analyses were performed using R software (version 4.3.1). Variables with more than 20% missing values were excluded from this study, and the remaining missing data were filled using multiple imputation. The missing percent of each variable is shown in Appendix A. Normally distributed continuous variables were presented as the mean ± standard deviation (SD), and comparisons between cohorts were made using the independent *t*-test. Non-normally distributed continuous variables were described as the median and interquartile range (IQR), with comparisons between cohorts conducted using the Wilcoxon rank sum test. Categorical variables were described as the frequency and percentage, and differences between cohorts were evaluated using the Chi-square test or Fisher’s exact test. A *p*-value < 0.05 was considered statistically significant.

Eligible DPUTIs were randomly divided into the training cohort (70%) and the validation cohort (30%). The training cohort was used to construct a nomogram, while the validation cohort was used for internal validation. Factor selection in the training cohort was performed using the LASSO regression algorithm. Subsequently, independent prognostic factors were determined using multivariate logistic regression. A nomogram was constructed based on the determined independent prognostic factors to predict the probability of sepsis occurrence in DPUTIs.

The nomogram was validated using discrimination, calibration curves, and DCA. The discrimination of the nomogram was assessed by multiple indicators, including the AUC, NRI, and IDI. The AUC of the nomogram was compared with SOFA and APACHE II scores. The optimal cutoff value, sensitivity, and specificity were determined based on the Youden index from the ROC curve. Furthermore, a calibration curve was plotted, and a Hosmer–Lemeshow test was conducted to evaluate the calibrating ability of the nomogram. Finally, the DCA curve was applied to describe the clinical utility of the nomogram.

## 3. Results

### 3.1. Statistical Characteristics

**Figure 1:**
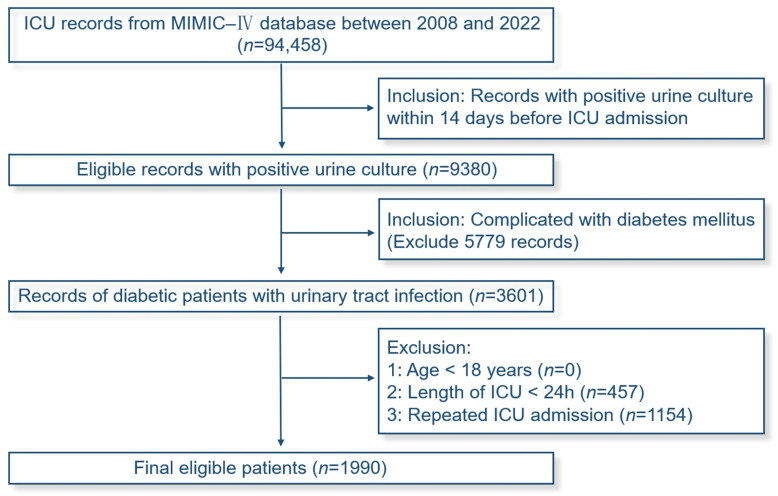
Flowchart of screening.

The patient selection flowchart based on the MIMIC-IV database is shown in Figure 1. After screening, a total of 94,458 ICU records were reviewed, and 1990 patients meeting the criteria were finally included. These 1990 patients were randomly divided into the training cohort (*n* = 1394) and the validation cohort (*n* = 596) in a 7:3 ratio. The baseline characteristics of the patients in the two cohorts are presented in Table 1. The training cohort was further divided into groups based on the occurrence of sepsis, with the baseline characteristics detailed in Appendix B.

### 3.2. LASSO and Logistic Regression Results

**Figure 2:**
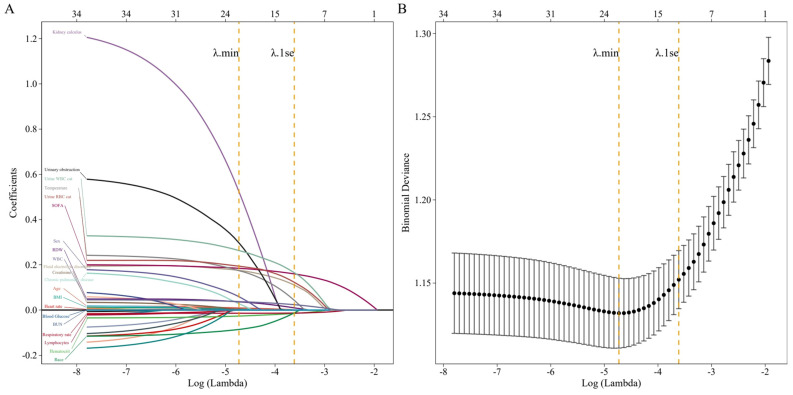
(**A**) illustrates the relationship between the variable coefficients and log(lambda) values. Each line corresponds to a different variable. As log(lambda) increases, the coefficients of the variables trend towards zero. (**B**) shows the relationship between Binomial Deviance and log(lambda). We plotted vertical lines at the optimal values using λ.min (left dashed line) and λ.1se (right dashed line, 1−SE standard). In this study, we selected the λ value according to the 1−SE criterion.

A total of 35 potential factors were included in this study, and factors were selected in the training group using the LASSO regression algorithm. After selection by LASSO regression, potential clinical factors were determined using a multivariate logistic regression model. Thirteen variables with non-zero coefficients were retained in the LASSO regression analysis (Figure 2A), among which four variables with no statistical significance (race, red blood cell distribution width, blood urea nitrogen, and fluid electrolyte disorders) were discarded after the multivariate logistic regression model, and nine variables with statistical significance were finally retained (Figure 2B). The results showed that the nine independent predictors of sepsis in diabetic patients with urinary tract infection included temperature, SOFA score, age, white blood cells, lymphocytes, hematocrit, blood glucose, urine RBC cat, and urine WBC cat. The corresponding OR values (95%CI) of each variable are shown in Table 2.

### 3.3. Building a Nomogram

**Figure 3:**
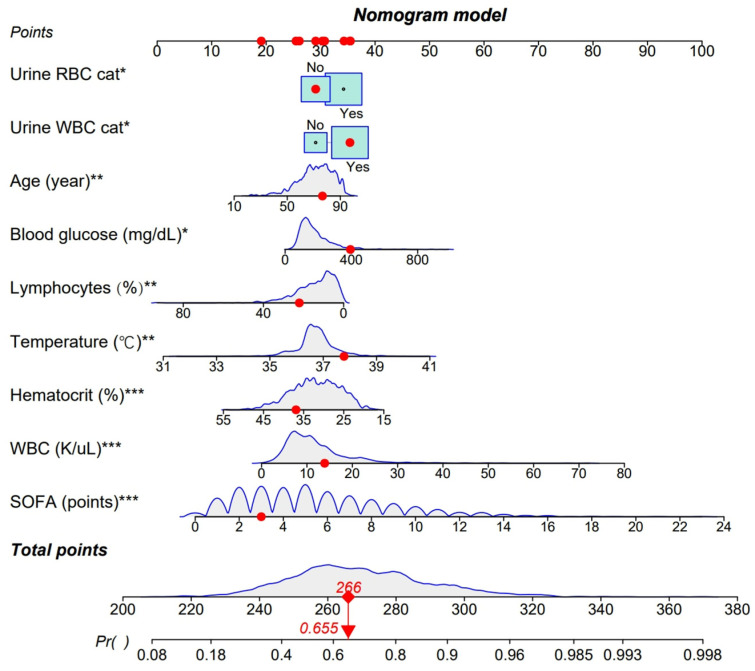
A nomogram based on lymphocytes, blood glucose, temperature, age, urine RBC cat, urine WBC cat, hematocrit, WBCs, and SOFA. * represents 0.01 < *p* < 0.05, ** represents *p* 0.001–0.01, and *** represents *p* < 0.001. An example of the application of the nomogram is shown above. The corresponding score of each variable is represented by a red dot. When the total score is 266 points, the probability of a DPUTI developing sepsis is 0.655.

The above nine variables were used to construct a nomogram to predict the probability of sepsis. For a particular patient, a vertical line is drawn from the corresponding value of each variable, and the corresponding score is represented by a red dot. Then, the scores are summed up to obtain a total score, which corresponds to a specific predicted probability of sepsis at the bottom of the nomogram. The higher the total score, the higher the risk of DPUTIs developing sepsis. The nomogram suggested that the higher the values of urine WBC cat, urine RBC cat, blood glucose, age, temperature, WBCs, and SOFA, the higher the total score. Lymphocytes and hematocrit were protective factors, and the higher the specific value, the lower the score. See Figure 3 for details.

### 3.4. Validation of the Nomogram

**Figure 4:**
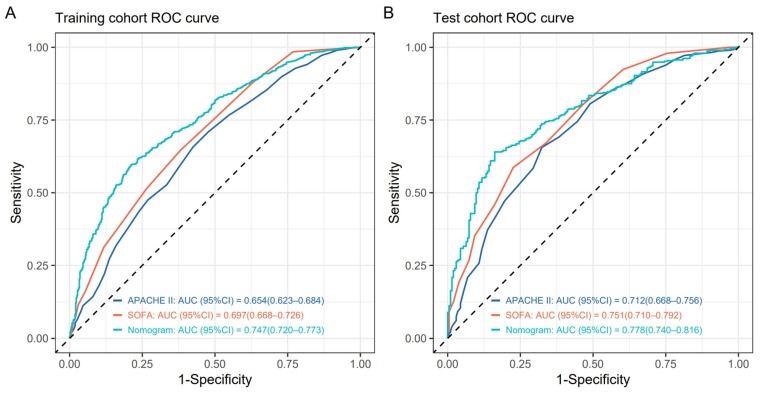
Performance of the predictive models for sepsis risk. (**A**,**B**) ROC curves of the nomogram, SOFA score, and APACHE Ⅱ score for predicting the likelihood of sepsis in DPUTIs. (**A**) is the training cohort; (**B**) is the test cohort. The dashed lines in (**A**,**B**) represent the baseline.

The discrimination of the nomogram was evaluated by comparing with the SOFA and APACHE Ⅱ score systems. The following results show that our nomogram has better discrimination:

1. AUC value: Figure 4 showed that the AUC values of the nomogram in the training cohort and the validation cohort were 0.747 (95%CI = 0.720–0.773) and 0.778 (95%CI = 0.740–0.816), respectively, which were higher than those of the SOFA and APACHE Ⅱ scores. In the training cohort, the optimal cutoff value of the nomogram was 0.698, and the sensitivity and specificity were 0.596 and 0.790, respectively. In the validation cohort, the optimal cutoff value was 0.688, the sensitivity was 0.640, and the specificity was 0.838. The corresponding AUC values, optimal cutoff values, sensitivity, and specificity of the SOFA and APACHE II scores in the training and validation cohorts are provided in Appendix C.
2. Comparison of NRI: Compared with the SOFA score, the NRI of the nomogram was 0.225 (95%CI = 0.170–0.280) in the training cohort and 0.205 (95%CI = 0.117–0.293) in the validation cohort. Compared with the APACHE Ⅱ score, the NRI of the nomogram was 0.265 (95%CI = 0.207–0.324) in the training cohort and 0.281 (95%CI = 0.184–0.378) in the validation cohort. See Table 3 for details.
3. Comparison of IDI: Compared with the SOFA score, the IDI of the nomogram was 0.067 (95%CI = 0.053–0.081) in the training cohort and 0.036 (95%CI = 0.009–0.063) in the validation cohort. Compared with the APACHE Ⅱ score, the IDI of the nomogram was 0.100 (95%CI = 0.084–0.117) in the training cohort and 0.085 (95%CI = 0.055–0.114) in the validation cohort. See Table 3 for details.

**Figure 5:**
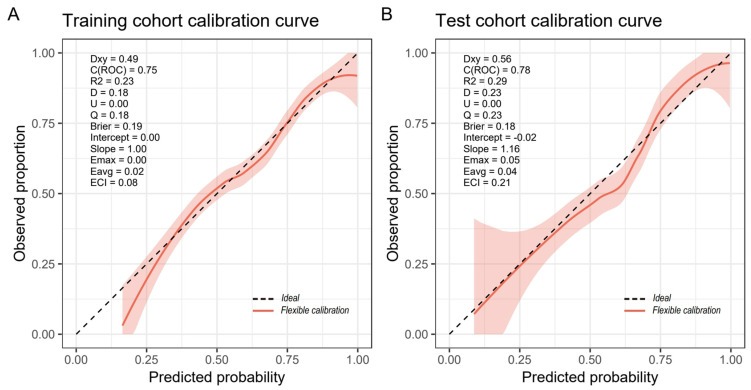
The calibration curves for the training cohort (**A**) and the validation cohort (**B**) indicated that the nomogram predictions were consistent with the actual observed outcomes. The Hosmer–Lemeshow test results suggested no statistical significance (both *p* > 0.05).

**Figure 6:**
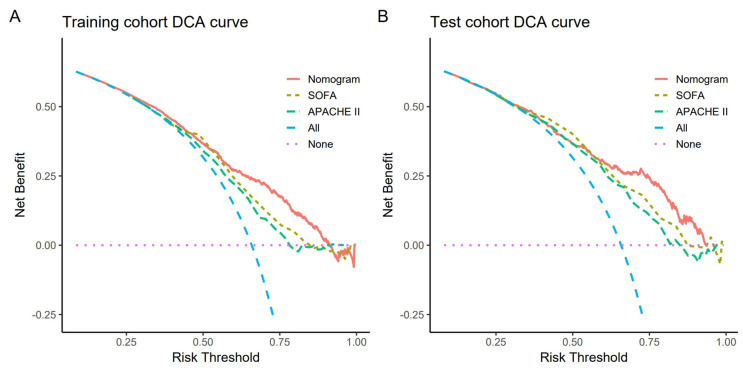
Decision curve analysis (DCA). DCA of the nomogram, SOFA score, and APACHE II score predicts the sepsis risk in DPUTIs. The pink dotted line represents the “treat−none” strategy, while the blue dashed line represents the “treat−all” strategy. (**A**) The training cohort; (**B**) the validation cohort.

Figure 5 shows the calibration curve of the nomogram, and the calibration curves of the training and validation cohorts suggested that the nomogram was consistent with the observed data results. The Hosmer–Lemeshow test results showed no statistical significance (training cohort: χ^2^ = 10.627, *p* = 0.224; validation group: χ^2^ = 11.070, *p* = 0.198). Finally, as shown in Figure 6, when the threshold probability of the two cohorts was between 0.25 and 0.9 in the DCA curve, the nomogram had good stability and consistency, and the overall net benefit of clinical intervention guided by the nomogram was higher than that of the SOFA and APACHE Ⅱ score systems.

## 4. Discussion

In this study, based on samples from the MIMIC-IV database, a nomogram model was developed to predict the risk probability of sepsis in DPUTIs and compared with commonly used disease severity scores. The nine independent predictors included in the model were as follows: urine RBC cat, urine WBC cat, blood glucose, age, temperature, WBCs, SOFA score, lymphocytes, and hematocrit. After comprehensive index evaluation, the model performed well and had good clinical utility.

This study shows that higher blood glucose levels are associated with a higher risk of sepsis. The most important feature of diabetes is hyperglycemia which cannot be effectively regulated autonomously [14]. Patients with poorly controlled hyperglycemia are prone to complicated infections and develop bacteremia more frequently, resulting in more hospitalizations [15] and severe complications such as urosepsis and ketoacidosis [16]. The worsening of infection causes severer glucose metabolism disorders, and blood glucose is higher in return [17]. There is a linear relationship between urine glucose and blood glucose levels [18]. Glucose is the energy source for most bacteria [19], and continuous positive urine glucose provides a good culture environment for specific bacteria to reproduce, which can make patients prone to UTI. The positive urine RBC cat test refers to hematuria. The presence of hematuria predicts structural damage to the urinary tract. The injury is easily complicated by infection, and the coexistence of blood ingredients and glucose in the urine may synergistically promote bacterial growth. Bacteria can easily penetrate the barrier of the urinary tract and invade the bloodstream, leading to sepsis, especially in Gram-negative bacilli [20]. Previous studies have found that when urinary tract stones cause obstruction, bacteria are more likely to reflux into the bloodstream, leading to sepsis [21]. However, this study investigated urinary obstruction as a variable and did not reach a consistent conclusion (*p* > 0.05), indicating the need for further research. Additionally, in this study, the risk of sepsis was significantly associated with an increase in urinary white blood cells. Pyuria is defined as >10^5^ white blood cells per milliliter [WBCs mL^−1^] urine [22]. When a urinary tract infection is present, white blood cells infiltrate the urine from the bloodstream, helping to clear the pathogenic infection and resulting in pyuria. A higher load of urinary white blood cells suggests a potentially more severe infection. The cytokines released by white blood cells regulate the duration and magnitude of the host’s inflammatory response, causing an imbalance between pro-inflammatory and anti-inflammatory reactions, which plays a role in the mechanism of sepsis [23].

The hematocrit refers to the proportion of red blood cells in whole blood. A decrease in the hematocrit indicates anemia [24]. One of the functions of red blood cells is to transport oxygen and carbon dioxide, supplying oxygen to tissue cells and removing carbon dioxide in the microcirculation [25]. When the hematocrit is too low, the total oxygen-carrying capacity of red blood cells is decreased, which also adversely affects oxygen supply and tissue repair from infection, causing organ damage that is difficult to repair. Guidelines from the Surviving Sepsis Campaign (2012) recommend that when the hematocrit is less than 0.24 (hemoglobin ≈ 8 g/dL), the transfusion of concentrated red blood cells is required to increase the hematocrit to 0.3 [26]. Still, guidelines from the Surviving Sepsis Campaign (2021) suggest that the hemoglobin concentration transfusion trigger is still recommended to be 7 g/dL [27]. Therefore, the hematocrit is a protective factor.

In most cases, when bacterial UTI occurs, the total count of white blood cells increases, indicating a significant inflammatory response. It is characterized by a rise in neutrophils (N), while the proportion of lymphocytes (L) decreases and N/L increases [28]. What is more, in early severe infection, the total lymphocyte count may decrease sharply, which is detrimental to infection control [29]. As the function of the immune response is inhibited by a severe inflammatory response, immune mechanisms such as the adhesion, chemotaxis, and phagocytosis of immune cells are inhibited. Sequentially, lymphocyte functions (both T and B lymphocytes) are decreased, which is not conducive to cellular immunity and humoral immunity [3]. The present study shows that the hematocrit and lymphocytes are protective factors against sepsis.

Persistently elevated body temperature is often indicative of an uncontrolled infection, as a result of pro-inflammatory factors. In the inflammatory response caused by infection, the brain’s response to body temperature originates from the stimulation of the thermoregulatory center by chemicals such as endogenous (like bacterial endotoxin) and exogenous pyrogens (like interleukin-6) [30,31]. The higher the concentration of these substances, the higher the body temperature may be, sometimes even more than 40 degrees Celsius. High fever increases heart rate and oxygen and energy consumption, all of which produce adverse effects on the human body [32].

An increase in age is associated with a more or less slow decline in cellular, systemic, and organ function. Once the mitochondrial and metabolic disorders in aging-related diseases are aggravated, sepsis is more likely to do harm to the targeted organs, like the heart, lungs, and kidneys [33], and the anti-attack ability is weakened. At the same time, while the immunity function of the elderly decreases, the risk of combined metabolic disorders and malignant diseases increases, and the risk of immune function damage is higher [34]. Therefore, the risk of infection and sepsis could increase with age.

Patients with sepsis are in severe condition and have a high risk of death. In the past, a variety of evaluation systems have been used to study disease severity and prognosis, and nomograms have also been established to predict the risk of urosepsis or death. Wei J et al. developed a prediction model for the risk of in-hospital mortality in elderly ICU patients with urosepsis [35]. Wang S et al. developed a nomogram for predicting the risk of sepsis in diabetic patients with urinary tract infection but mainly focused on urine culture analysis, C-reactive protein, white blood cells, and albumin [36]. In our study, a nomogram was developed using the SOFA score in combination with eight other risk factors. The combined indicators are easy to obtain and calculate, which can improve the accuracy of sepsis prediction and facilitate the early assessment of sepsis risk prior to or after hospital admission. The prediction model can be applied to clinical practice for the management of DPUTIs, assessing the risk of sepsis and determining whether patients should receive early intervention or intensive care.

The data of this study were extracted from the MIMIC database, which is reliable and representative. The nomogram developed in this study can be put into practice in community hospitals to facilitate early interventions and may help to reduce the incidence of sepsis in DPUTIs. However, this study has limitations, as it is retrospective and single-centered and has a relatively small sample size. Further multiple-centered studies should be conducted for external validation and improvement in clinical utility. In addition, other factors were not included. Inflammatory biomarkers like procalcitonin were absent. More than 20% of records had missing glycated hemoglobin (HbA1c) values. Hence, we failed to assess the average blood glucose levels 90 days before the onset of sepsis.

## 5. Conclusions

The nomogram established in this study helps clinicians predict the probability of sepsis in DPUTIs. For patients with diabetes complicated by urinary tract infection, a higher nomogram score corresponds to a greater risk of developing sepsis. Clinically, it is necessary to dynamically monitor the function of vital organs in patients with high scores to assess whether they have developed sepsis. Some patients may even require early intensified treatment in advance. This study provides evidence for optimizing the management of related risk factors.

## Figures and Tables

**Table 1:** Patient characteristics.

Variables	Training Cohort	Validation Cohort	*t*/χ^2^/*Z*	*p*-Value
*n*	1394	596		
Sepsis, *n* (%)			<0.001	>0.999
No	476 (34.15)	204 (34.23)		
Yes	918 (65.85)	392 (65.77)		
Age (years)	71.40 ± 13.35	71.24 ± 13.16	0.246	0.805
Sex, *n* (%)			0.058	0.810
Female	798 (57.25)	337 (56.54)		
Male	596 (42.75)	259 (43.46)		
Race, *n* (%)			0.352	0.950
White	838 (60.11)	361 (60.57)		
Black	251 (18.01)	101 (16.95)		
Other	305 (21.88)	134 (22.48)		
BMI (kg/m^2^)	30.71 ± 8.54	30.01 ± 8.42	1.690	0.091
Vital signs				
Heart rate (bpm)	89.98 ± 20.07	90.15 ± 20.14	−0.175	0.861
Mean arterial pressure (mmHg)	82.21 ± 18.87	80.11 ± 18.10	2.350	0.019
Respiratory rate (insp/min)	19.58 ± 6.05	19.48 ± 5.90	0.343	0.732
Temperature (°C)	36.72 ± 0.78	36.70 ± 0.90	0.370	0.711
Laboratory indicators				
WBCs (K/μL)	10.30 (7.30; 14.40)	10.25 (6.90; 14.25)	429,904	0.217
Neutrophils (%)	78.40 (69.80; 86)	77.55 (67.90; 85.45)	437,257	0.063
Lymphocytes (%)	11.90 (6.60; 19.30)	13.05 (7.05; 20.95)	391,385	0.041
Hematocrit (%)	31.99 ± 6.11	31.74 ± 6.43	0.824	0.410
Platelets (K/μL)	206.00 (145.00; 278.00)	199.00 (150.00; 275.50)	418,576	0.788
RDW (%)	15.63 ± 2.48	15.77 ± 2.58	−1.100	0.272
Blood glucose (mg/dL)	155.00 (115.00; 219.00)	155.00 (118.50; 215.00)	414,908	0.966
BUN (mg/dL)	28.00 (17.00; 47.00)	28.00 (17.00; 47.25)	409,702	0.627
Creatinine (mg/dL)	1.20 (0.90; 2.10)	1.30 (0.90; 2.00)	408,037	0.529
Bilirubin total (mg/dL)	0.50 (0.30; 0.80)	0.50 (0.30; 0.85)	410,851	0.696
Urine RBCs (#/hpf)	5.00 (1.00; 26.00)	6.50 (1.00; 34.50)	397,674	0.129
Urine WBCs (#/hpf)	28.00 (4.00; 145.00)	33.00 (6.00; 159.00)	403,358	0.302
Urine PH	6.00 (5.50; 6.50)	6.00 (5.50; 6.50)	424,412	0.433
Urine ketone, *n* (%)			0.420	0.517
Negative	1096 (78.62)	477 (80.03)		
Positive	298 (21.38)	119 (19.97)		
Urine protein, *n* (%)			0.405	0.525
Negative	395 (28.34)	178 (29.87)		
Positive	999 (71.66)	418 (70.13)		
Urine RBC cat, *n* (%)			6.950	0.008
No	535 (38.38)	191 (32.05)		
Yes	859 (61.62)	405 (67.95)		
Urine WBC cat, *n* (%)			2.690	0.101
No	388 (27.83)	144 (24.16)		
Yes	1006 (72.17)	452 (75.84)		
Comorbid disease, *n* (%)				
Hypertension, *n* (%)			0.303	0.582
No	269 (19.30)	108 (18.12)		
Yes	1125 (80.70)	488 (81.88)		
Congestive heart failure, *n* (%)			0.169	0.681
No	782 (56.10)	341 (57.21)		
Yes	612 (43.90)	255 (42.79)		
Chronic pulmonary disease, *n* (%)			3.030	0.082
No	976 (70.01)	441 (73.99)		
Yes	418 (29.99)	155 (26.01)		
Liver disease, *n* (%)			1.530	0.216
No	1190 (85.37)	522 (87.58)		
Yes	204 (14.63)	74 (12.42)		
Renal disease, *n* (%)			0.084	0.772
No	851 (61.05)	359 (60.23)		
Yes	543 (38.95)	237 (39.77)		
Cerebrovascular disease, *n* (%)			0.329	0.566
No	1122 (80.49)	487 (81.71)		
Yes	272 (19.51)	109 (18.29)		
Paraplegia, *n* (%)			2.490	0.114
No	1312 (94.12)	572 (95.97)		
Yes	82 (5.88)	24 (4.03)		
Urinary obstruction, *n* (%)			0.896	0.344
No	1347 (96.63)	570 (95.64)		
Yes	47 (3.37)	26 (4.36)		
Fluid electrolyte disorders, *n* (%)			<0.001	>0.999
No	572 (41.03)	245 (41.11)		
Yes	822 (58.97)	351 (58.89)		
Cancer, *n* (%)			0.637	0.425
No	1231 (88.31)	518 (86.91)		
Yes	163 (11.69)	78 (13.09)		
Kidney calculus, *n* (%)			2.170	0.141
No	1372 (98.42)	580 (97.32)		
Yes	22 (1.58)	16 (2.68)		
Disease severity score (points)				
APACHE Ⅱ	23.00 (18.00; 28.00)	23.00 (19.00; 28.00)	406,805	0.463
SOFA	5.00 (3.00; 7.00)	5.00 (3.00; 8.00)	404,092	0.333

BMI refers to body mass index, WBC refers to white blood cell, RDW refers to red blood cell distribution width, BUN refers to blood urea nitrogen, RBC refers to red blood cell, urine RBC/WBC cat refers to urine red/white blood cell classification, APACHE Ⅱ refers to acute physiology and chronic health evaluation Ⅱ, SOFA refers to sequential organ failure assessment, and #/hpf refers to cell counts per high power field.

**Table 2:** Independent risk factors for sepsis in DPUTIs in the multivariate logistic analysis.

Factors	OR	95%CI	*p*-Value
SOFA (points)	1.242	1.189, 1.300	<0.001
Age (years)	1.013	1.004, 1.023	0.006
Temperature (°C)	1.300	1.098, 1.541	0.002
WBCs (K/μL)	1.046	1.023, 1.070	<0.001
Lymphocytes (%)	0.980	0.968, 0.993	0.002
Hematocrit (%)	0.961	0.942, 0.981	<0.001
Blood glucose (mg/dL)	1.002	1.000, 1.003	0.013
Urine RBC cat (Yes vs. No)	1.316	1.007, 1.718	0.044
Urine WBC cat (Yes vs. No)	1.402	1.052, 1.866	0.021

Predictors were derived from a multiple logistic regression model based on the 13 features selected from the LASSO regression model. Four variables without statistical significance—race, RDW, BUN, and fluid electrolyte disorders—were discarded, ultimately retaining nine variables with statistical significance. Results for independent predictors are presented as odds ratios and 95% confidence intervals (CIs). SOFA refers to sequential organ failure assessment (min: 0; max: 24), WBC refers to white blood cell, urine RBC/WBC cat refers to urine red/white blood cell classification, RDW refers to red blood cell distribution width, BUN refers to blood urea nitrogen, OR refers to odds ratio, and CI refers to confidence interval.

**Table 3:** NRI/IDI outcomes of comparison between nomogram and SOFA/APACHE Ⅱ score systems in both training cohort and validation cohort.

Score System	Nomogram NRI (95%CI)		Nomogram IDI (95%CI)	
	Training Cohort	Validation Cohort	Training Cohort	Validation Cohort
SOFA	0.225 (0.170–0.280)	0.205 (0.117–0.293)	0.067 (0.053–0.081)	0.036 (0.009–0.063)
APACHE Ⅱ	0.265 (0.207–0.324)	0.281 (0.184–0.378)	0.100 (0.084–0.117)	0.085 (0.055–0.114)

**Figure A1:**
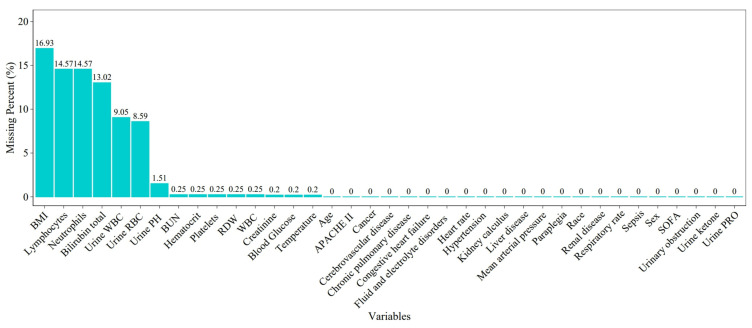
Missing percent of each variable.

## Data Availability

The data that support the findings of this study are openly available on the MIMIC-IV website at https://physionet.org/content/mimic iv/3.0/, accessed on 1 September 2024.

## Appendix B

**Table A1:** Characteristics of non-sepsis/sepsis subgroups in the training cohort.

Variables	Non-Sepsis	Sepsis	*t*/χ^2^/*Z*	*p*-Value
*n*	476	918		
Age (years)	69.85 ± 14.47	72.20 ± 12.66	−3.010	0.003
Sex, *n* (%)			3.770	0.052
Female	290 (60.92)	508 (55.34)		
Male	186 (39.08)	410 (44.66)		
Race, *n* (%)			10.000	0.018
White	263 (55.25)	575 (62.64)		
Black	91 (19.12)	160 (17.43)		
Other	122 (25.63)	183 (19.94)		
BMI (kg/m^2^)	30.56 ± 8.65	30.79 ± 8.48	−0.485	0.628
Vital signs				
Heart rate (bpm)	88.02 ± 19.24	91.00 ± 20.43	−2.680	0.007
Mean arterial pressure (mmHg)	83.71 ± 17.98	81.43 ± 19.28	2.190	0.029
Respiratory rate (insp/min)	19.09 ± 5.98	19.83 ± 6.08	−2.190	0.029
Temperature (°C)	36.64 ± 0.66	36.75 ± 0.83	−2.670	0.008
Laboratory indicators				
WBCs (K/μL)	9.20 (6.80; 12.10)	11.00 (7.50; 15.50)	174,010	<0.001
Neutrophils (%)	75.30 (67.90; 82.80)	80.30 (71.00; 86.90)	176,106	<0.001
Lymphocytes (%)	15.30 (9.00; 21.80)	10.00 (5.70; 17.40)	276,569	<0.001
Hematocrit (%)	33.10 ± 6.05	31.42 ± 6.06	4.900	<0.001
Platelets (K/μL)	212.50 (158.00; 279.00)	201.00 (139.00; 277.00)	233,955	0.030
RDW (%)	15.13 ± 2.52	15.90 ± 2.41	−5.500	<0.001
Blood glucose (mg/dL)	145.50 (110.00; 199.50)	163.00 (118.00; 232.00)	194,258	<0.001
BUN (mg/dL)	23.00 (15.00; 38.00)	31.00 (19.00; 52.00)	168,204	<0.001
Creatinine (mg/dL)	1.00 (0.80; 1.50)	1.40 (1.00; 2.30)	160,764	<0.001
Bilirubin total (mg/dL)	0.40 (0.30; 0.70)	0.50 (0.30; 0.90)	197,485	0.003
Urine RBCs (#/hpf)	3.00 (1.00; 15.00)	7.00 (1.00; 35.00)	179,752	<0.001
Urine WBCs (#/hpf)	14.00 (2.00; 64.00)	47.00 (8.00; 182.00)	166,186	<0.001
Urine PH	6.00 (5.50; 6.50)	6.00 (5.50; 6.50)	227,836	0.180
Urine ketone, *n* (%)			0.863	0.353
Negative	367 (77.10)	729 (79.41)		
Positive	109 (22.90)	189 (20.59)		
Urine protein, *n* (%)			12.900	<0.001
Negative	164 (34.45)	231 (25.16)		
Positive	312 (65.55)	687 (74.84)		
Urine RBC cat, *n* (%)			27.100	<0.001
No	228 (47.90)	307 (33.44)		
Yes	248 (52.10)	611 (66.56)		
Urine WBC cat, *n* (%)			25.400	<0.001
No	173 (36.34)	215 (23.42)		
Yes	303 (63.66)	703 (76.58)		
Comorbid disease, *n* (%)				
Hypertension, *n* (%)			0.230	0.631
No	88 (18.49)	181 (19.72)		
Yes	388 (81.51)	737 (80.28)		
Congestive heart failure, *n* (%)			1.160	0.281
No	277 (58.19)	505 (55.01)		
Yes	199 (41.81)	413 (44.99)		
Chronic pulmonary disease, *n* (%)			1.300	0.255
No	343 (72.06)	633 (68.95)		
Yes	133 (27.94)	285 (31.05)		
Liver disease, *n* (%)			10.400	0.001
No	427 (89.71)	763 (83.12)		
Yes	49 (10.29)	155 (16.88)		
Renal disease, *n* (%)			6.440	0.011
No	313 (65.76)	538 (58.61)		
Yes	163 (34.24)	380 (41.39)		
Cerebrovascular disease, *n* (%)			1.510	0.219
No	374 (78.57)	748 (81.48)		
Yes	102 (21.43)	170 (18.52)		
Paraplegia, *n* (%)			0.130	0.719
No	446 (93.70)	866 (94.34)		
Yes	30 (6.30)	52 (5.66)		
Urinary obstruction, *n* (%)			7.160	0.007
No	469 (98.53)	878 (95.64)		
Yes	7 (1.47)	40 (4.36)		
Fluid electrolyte disorders, *n* (%)			33.200	<0.001
No	246 (51.68)	326 (35.51)		
Yes	230 (48.32)	592 (64.49)		
Cancer, *n* (%)			1.170	0.279
No	427 (89.71)	804 (87.58)		
Yes	49 (10.29)	114 (12.42)		
Kidney calculus, *n* (%)			5.160	0.023
No	474 (99.58)	898 (97.82)		
Yes	2 (0.42)	20 (2.18)		
Disease severity score (points)				
APACHE Ⅱ	20.00 (16.00; 25.00)	24.00 (20.00; 29.00)	151332	<0.001
SOFA	4.00 (2.00; 6.00)	6.00 (4.00; 8.00)	132,494	<0.001

BMI refers to body mass index, WBC refers white blood cell, RDW refers to red blood cell distribution width, BUN refers to blood urea nitrogen, RBC refers to red blood cell, urine RBC/WBC cat refers to urine red/white blood cell classification, APACHE Ⅱ refers to acute physiology and chronic health evaluation Ⅱ, SOFA refers to sequential organ failure assessment, and #/hpf refers to cell counts per high power field.

## Appendix C

**Table A2:** AUC, cutoff, sensitivity, and specificity outcomes of comparison between nomogram and SOFA/APACHE Ⅱ score systems in both training cohort and testing cohort.

Cohort	Nomogram and Disease Severity Score		
	Nomogram	SOFA	APACHE Ⅱ
**Training cohort**			
AUC	0.747 (0.720–0.773)	0.697 (0.668–0.726)	0.654 (0.623–0.684)
Cutoff value	0.698	4.5	21.5
Sensitivity	0.596	0.647	0.657
Specificity	0.790	0.618	0.576
**Testing cohort**			
AUC	0.778 (0.740–0.816)	0.751 (0.710–0.792)	0.712 (0.668–0.756)
Cutoff value	0.688	5.5	22.5
Sensitivity	0.640	0.587	0.656
Specificity	0.838	0.775	0.676
